# Whole-genome resequencing reveals chromosomal fusion-driven early stages of XY chromosomes evolution in the darkbarbel catfish (*Tachysurus vachellii*)

**DOI:** 10.1186/s12983-025-00588-w

**Published:** 2025-11-18

**Authors:** Jianjun Liu, Min Tang, Guoqing Duan, Huan Wang, Siqi Liu, Liuwang Nie, Huaxing Zhou

**Affiliations:** 1 Anhui Key Laboratory of Aquaculture & Stock Enhancement, Fisheries Research Institution, Anhui Academy of Agricultural Sciences, Hefei, China; 2 The Anhui Provincial Key Laboratory of Biodiversity Conservation and Ecological Security in the Yangtze River Basin, Life Science College of Anhui Normal University, Wuhu, Anhui China; 3 Jiangxi Provincial Key Laboratory of Conservation Biology, College of Forestry, Jiangxi Agricultural University, Nanchang, 330045 China

**Keywords:** *Tachysurus vachellii*, Sex chromosome, Evolution, Linkage disequilibrium

## Abstract

This study investigates the early stages of putative sex chromosome evolution in the darkbarbel catfish (*Tachysurus vachellii*) through whole-genome resequencing of 31 individuals. We identified the sex-determining region (SDR) on chromosome 3 (chr3), with an *F*_ST_ value of 0.5, indicating the presence of relatively putative young XY sex chromosomes. Our analyses revealed significant genetic differentiation between male and female groups, suggesting that these young sex chromosomes contribute to observed genetic divergence. Notably, among SDR, pseudoautosomal region (PAR), and autosomes, PAR exhibits the highest synonymous substitutions (*d*_S_) values. And the significantly reduced ratio of nonsynonymous-to-synonymous substitutions (*d*_N_/*d*_S_) in SDR demonstrates intense purifying selection acting on this region, and the progressive *d*_N_/*d*_S_ gradient (SDR < PAR < autosomes) reveals an incipient differentiation continuum from PAR to SDR. Our findings provide valuable insights into the dynamics of sex chromosome evolution in fish and underscore the need for further research to explore these mechanisms.

**Supplementary Information:**

The online version contains supplementary material available at 10.1186/s12983-025-00588-w.

## Introduction

**Fig. 1:**
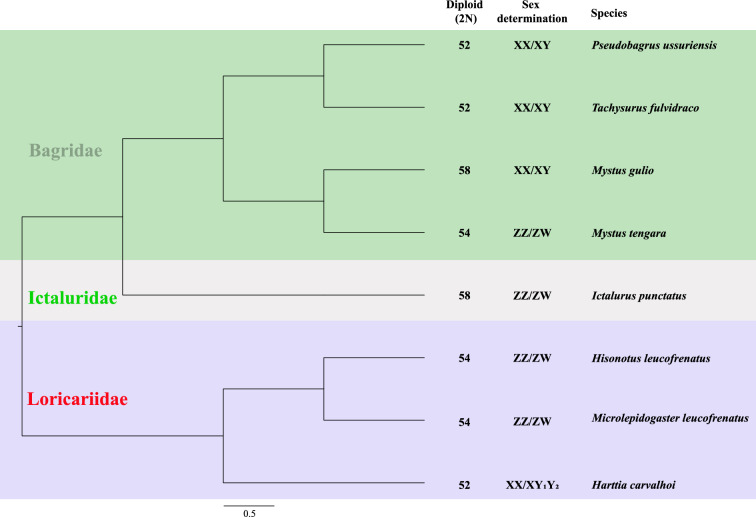
Sex chromosomes in Siluriformes The tree structure is derived from taxonomic information provided by Timetree (http://www.timetree.org/); sex-chromosome data are from Tree of Sex (https://www.treeofsex.org/).

Sex chromosomes play a crucial role in defining sexual dimorphism and reproductive strategies in many species [1–3]. In teleost fishes, the evolution of sex chromosomes is particularly dynamic, often characterized by rapid changes in chromosomal structure and function [4, 5]. In Siluriformes, karyotype numbers and sex chromosome evolution exhibit complex patterns. Within Bagridae, both XY and ZW systems are present, with even congeneric species (*Mystus*) displaying both systems [6]. Notably, closely related species (*Pseudobagrus ussuriensis* and *Tachysurus fulvidraco*) show conserved XY systems with identical chromosome numbers (Fig. 1) [7, 8]. Understanding the evolutionary trajectory of these chromosomes can provide insights into the mechanisms of sex determination and the factors driving genetic divergence between sexes. The darkbarbel catfish (*T. vachellii*) serves as an ideal model for studying these processes due to its relatively recent divergence from closely related species, such as the yellow catfish (*T. fulvidraco*), which exhibit young XY sex chromosomes [8].

Recent advancements in genomic technologies have enabled whole-genome resequencing, facilitating comprehensive analyses of genetic variations, including single nucleotide polymorphisms (SNPs) and chromosomal structures [9–11]. This study aims to delineate the sex-determining region (SDR) in the darkbarbel catfish, employing fixation-index (*F*_ST_) analyses to detect regions of genetic divergence between male and female individuals. We hypothesize that the XY sex chromosomes in this species are in the initial stages of differentiation, potentially influenced by recent chromosomal fusion events that may have contributed to the formation of novel sex-determining loci.

Furthermore, we explore the implications of linkage disequilibrium (LD) and the relationship between chromosomal fusion and the evolutionary dynamics of sex chromosomes. By integrating analyses of synonymous (*d*_S_) and nonsynonymous (*d*_N_) mutations, we assess the selective pressures acting on these chromosomes, comparing them to autosomes to better understand their evolutionary trajectories. Our findings aim to elucidate the processes governing the evolution of sex chromosomes in fish and contribute to the broader understanding of genetic mechanisms underlying sexual differentiation.

## Results

### Identification of putative young XY chromosomes

**Fig. 2:**
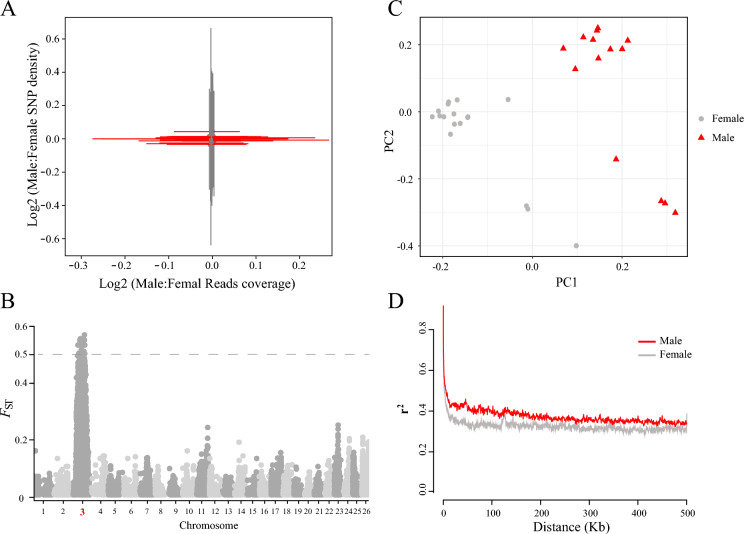
Identification of young XY chromosomes in the darkbarbel catfish **A** Analysis of reads coverage and SNP density. **B** Analysis of *F*_ST_ revealed young chromosome of chr3**. C** PCA principal component analysis based on chr3. **D** LD decay of male and female individuals based on chr3.

High-depth whole-genome resequencing of the 31 samples of the darkbarbel catfish generated a total of approximately 491.31 Gb raw data with mean Q20 >  = 95% and an average depth of 18 ~ 26 × per individual. After filtering, approximately 479.54 Gb clean data were retained for subsequent reads mapping and SNP calling (Supplemental table S1). To investigate whether there was sex chromosome divergence in the darkbarbel catfish, the clean reads of 13 male and 18 female offspring individuals were mapped to the public darkbarbel catfish genome (GCA_033026395.1) to identify chromosomes of reads coverage and SNP density between sexes. In the male heterogametic sex determination system, recombination-suppressed regions in females (XX) have reduced mapping efficiency relative to the male (XY) genome assembly, and SNPs in the regions also result in lower average male heterozygosity in the absence of homomorphic sex chromosomes [12, 13]. In this study, the read coverage and SNP density of the darkbarbel catfish were plotted together. However, none of the chromosome values show signals from the sex chromosomes (Fig. 2A). Given the numerous studies reported previously [14, 15], many fish species have young sex chromosomes. We used VCFtools software to calculate the fixation-index (*F*_ST_) to identify the differentiated regions of the sex chromosomes. In this study, when there were more than 10 male and female samples, the *F*_ST_ value of 0.5 was suggested to be considered as the differentiated region of the sex chromosomes [16]. Our results show that only the 12 Mb ~ 30 Mb region of chromosome 3 (chr3) has an *F*_ST_ value of 0.5 among all chromosomes (Fig. 2B, Supplemental Figure S1). The combined results indicate that the chr3 is a putative young sex chromosome in the darkbarbel catfish.

We selected the SNPs of chr3 from the darkbarbel catfish for the principal component analysis (PCA) and Linkage Disequilibrium (LD) decay analysis. Our results indicate significant genetic differentiation between the male and female groups (Fig. 2C), suggesting that the putative young sex chromosomes contribute to the observed genetic divergence. Our results revealed that the LD decay in the male population was significantly higher than that in the female population, as indicated by the greater r^2^ values (Fig. 2D). This observation suggests that the genetic architecture of the sex chromosomes may be more complex in males, consistent with an XY sex determination system. Therefore, we infer that the presence of young XY sex chromosomes is likely responsible for the observed patterns of genetic differentiation.

## Chromosomal fusion drives the independent origin of putative sex chromosomes

**Fig. 3:**
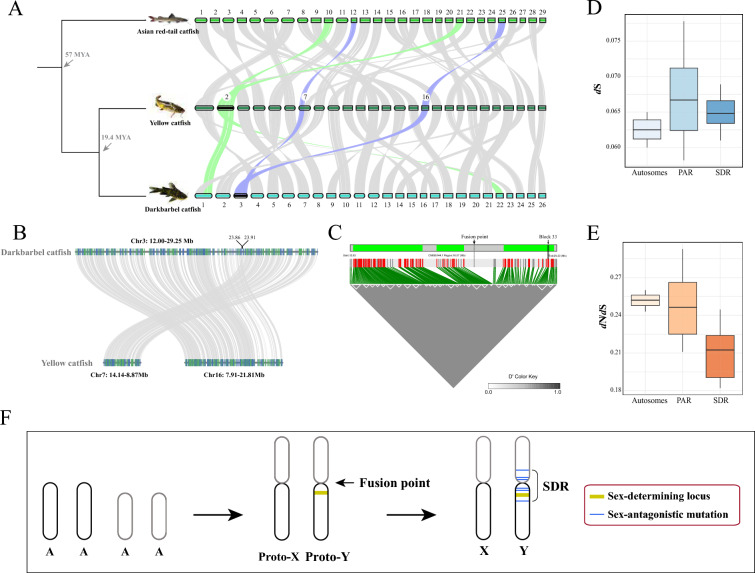
Chromosomal fusion-driven evolution of putative young XY chromosomes **A** Pairwise whole-genome alignments across three chromosome-level assemblies of the Asian red-tail catfish, the darkbarbel catfish and the yellow catfish. The black-labeled chromosomes in the figure represent putative sex chromosomes, such as Chr2 in Yellow catfish and Chr3 in darkbarbel catfish, as indicated. **B** A local microsynteny analysis of Chr3 (12.00-29.25 Mb) of the darkbarbel catfish and Chr7 (8.87–14.14 Mb) and Chr16 (7.91–21.81 Mb) of the yellow catfish. **C** Heatmap of LD on part of chr3. The white triangular boxes indicate LD blocks, the gray bar above represents the 12.62 Mb to 29.20 Mb region of Chr3, and the green boxes denote three clustered LD regions, respectively. The dashed line at the fusion point represents the potential chromosomal fusion site between the two chromosomes. **D** *d*_S_ values for *T. vachellii* and *T. fulvidraco* of PAR and SDR in sex chromosome, and autosomes. **E** *d*_N_/*d*_S_ values for *T. vachellii* and *T. fulvidraco* of PAR and SDR in sex chromosome, and autosomes. **F** The origin and evolution of homomorphic young sex chromosomes via autosome–autosome fusion in the darkbarbel catfish.

To analyze the origin of putative sex chromosomes in the darkbarbel catfish, we selected two chromosome-level assembled genomes: the Asian red-tail catfish (*Hemibagrus wyckioides*), and the yellow catfish (*T. fulvidraco*). The chromosome 2 (chr2) was presumed to be the putative young X/Y chromosome in the yellow catfish [8], while no sex chromosomes have been reported to date in the Asian red-tail catfish. The darkbarbel catfish exhibits a closer phylogenetic relationship with the yellow catfish, with a divergence time of approximately 19.4 million years ago (MYA), whereas it has a more distant relationship with the Asian red-tail catfish, which diverged around 94 MYA [17]. The synteny analysis reveals that the chr3 of the darkbarbel catfish is homologous to chr7 and chr16 of the yellow catfish, as well as to the chr12 and chr25 of the Asian red-tail catfish. Additionally, the chr2 (sex chromosome) of the yellow catfish is homologous to chr1 and chr22 of the darkbarbel catfish, as well as to the chr10 and chr21 of the Asian red-tail catfish (Fig. 3A). We also performed a microsynteny analysis on the Chr3 chromosome (12.00–29.25 Mb) of the darkbarbel catfish and Chr7 (8.87–14.14 Mb) and Chr16 (7.91–21.81 Mb) of the yellow catfish. The results revealed that the fusion site region on Chr3 of the darkbarbel catfish is located at 23.86–23.91 Mb (Fig. 3B). Although both the darkbarbel catfish and the yellow catfish possess young XY sex chromosomes, their sex chromosomes did not evolve by descent. Moreover, these two distinct young sex chromosomes appear to have undergone chromosomal fusion. These results suggest that the putative sex chromosomes of the darkbarbel catfish and the yellow catfish originated independently during later stages of evolution and chromosomal fusion may be a driving force in the evolution of sex chromosomes.

We extracted SNPs from chr3, where males are heterozygous and females are homozygous. Linkage disequilibrium analysis revealed that there are 219 SNPs with strong linkage in the region of 12.62 Mb to 29.20 Mb on chr3 (Fig. 3C). Notably, we previously identified a sex-differentiated region based on *F*_ST_ analysis that spans 12 Mb to 30 Mb (Supplemental Figure S1), which aligns closely with the linkage disequilibrium region. Therefore, we propose that the region spanning 12.62–29.20 Mb represents the SDR, while the remaining regions constitute pseudoautosomal region (PAR) in chr3. Furthermore, these linked loci formed 34 distinct blocks, which appear to be distributed across three regions within the sex differentiation area: 12.91 Mb to 18.44 Mb, 19.59 Mb to 21.75 Mb, and 24.91 Mb to 28.92 Mb. The largest LD block, designated as Block 33, spans from 17.93 Mb to 19.22 Mb and contains a total of 12 LD loci. This region includes seven genes, among which are Serine/threonine-protein kinase 35 (*STK35*) and Forkhead box protein O6 (*FOXO6*). *STK35* has previously been shown to be highly expressed in human testes [18], while *FOXO6* is highly expressed in the testes of *Spinibarbus hollandi* [19]. Interestingly, chr3 originated from a fusion event, and we found that the fusion point lies precisely within the sex chromosome differentiation region, suggesting that the sex-determining region spans the fusion site, which may suggest some association between the differentiation of fish sex chromosomes and chromosomal fusion events.

## Estimates of synonymous and nonsynonymous mutation

In the darkbarbel catfish, we performed genome-wide calculations of synonymous substitution rates (*d*_S_) and the ratio of nonsynonymous-to-synonymous substitutions (*d*_N_/*d*_S_) for all protein-coding genes across three genomic compartments: autosomes, SDR, and PAR. Comparative analysis revealed distinct evolutionary patterns among these regions. For values of *d*_S_, autosomes exhibited the lowest mean evolutionary rate (0.0625; 95% CI: 0.0624–0.0626), followed by the SDR (0.0650; 95% CI: 0.0643–0.0658), with PAR showing the highest *d*_S_ values (0.0672; 95% CI: 0.0660–0.0685) (Fig. 3D). Conversely, selection pressure analysis (*d*_N_/*d*_S_) demonstrated an inverse pattern: autosomes displayed the highest mean* d*_N_/*d*_S_ (0.2519; 95% CI: 0.2512–0.2525), followed by PAR (0.2479; 95% CI: 0.2394–0.2564), while the SDR showed significantly lower values (0.2084; 95% CI: 0.2016–0.2152) (Fig. 3E). One-way ANOVA confirmed statistically significant differences among all three genomic regions for both *d*_S_ and *d*_N_/*d*_S_ measurements (*p* < 0.0001 for all comparisons), highlighting distinct evolutionary constraints operating on these functionally distinct chromosomal segments.

## Discussion

In this study, we conducted an analysis of putative sex chromosomes by constructing sibling families that produced 18 female offspring and 13 male offspring. This approach eliminates noise from individual or interpopulation trait differences. Using the justified *F*_ST_ analysis method, we identified the region from 12 to 30 Mb on chr3 of the darkbarbel catfish as the sex-determining region (SDR), setting the *F*_ST_ difference between male and female populations at a threshold of 0.5. Analysis of read coverage and SNP density reveals no significant differences between male and female individuals, indicating that the sex chromosomes of the darkbarbel catfish are relatively young. We propose that the putative XY sex chromosomes may be in the initial stages of differentiation. The significantly reduced *d*_N_/*d*_S_ ratio in the SDR (0.2084) demonstrates intense purifying selection acting on this region, and the progressive *d*_N_/*d*_S_ gradient (SDR < PAR < autosomes) reveals an incipient differentiation continuum from PAR to SDR, offering critical empirical support for characterizing the dynamic evolutionary trajectories during initial sex chromosome formation.

The darkbarbel catfish and the yellow catfish are closely related species with a short divergence time. Although they have the same number of chromosomes, three chromosomal fusion and fission events have been identified between them (Fig. 3A). Interestingly, two of these events led to the independent origins of young XY sex chromosomes in each species, with no structural sequence similarity between the two sex chromosomes. In fish, many studies have documented the formation of established sex chromosomes (such as XY) and events involving the fusion of autosomes to create neo-sex chromosomes (Y-A fusion) [20]. However, in this study, we propose that the origins of the sex chromosomes in the darkbarbel catfish and the yellow catfish resulted from the fusion of two pairs of autosomes, which subsequently acquired sex-determining loci. This process likely caused a linkage disequilibrium effect, leading to the gradual evolution of the sex chromosomes (Fig. 3F). We suggest that chromosomal fusion may increase the probability of sex chromosome formation and could be one of the driving forces behind sex chromosome evolution. However, more data is needed to support this view.

## Conclusion

In conclusion, our study sheds light on the early stages of putative sex chromosome evolution in the darkbarbel catfish. By analyzing sibling families and employing *F*_ST_ analysis, we identified a sex differentiation region on chr3, indicating the presence of relatively putative young XY sex chromosomes. We propose chromosomal fusion may drive sex chromosome evolution. Our findings enhance the understanding of sex chromosome dynamics in fish and highlight the need for further research to validate these insights.

## Materials and methods

### Sampling and sequencing

A mature female and a mature male *T. vachellii* fish were selected from a farm for isolated breeding. One year later, 31 offspring were produced. Each offspring was dissected, and the sex of the individuals was determined by observing the gonads, resulting in the identification of 18 female offspring and 13 male offspring. All samples were collected and stored immediately in a -80 °C freezer prior to DNA extraction.

Genomic DNA of each individual was extracted from *T. vachellii* using the E.Z.N.A. Tissue DNA kit (Omega Bio-Tek) following the Tissue DNA—Spin Protocol. After the DNA samples were delivered, a quality control test was carried out on the specimens, and the qualified DNA (> 3 µg; concentration > 30 ng/µl; OD260 /OD280 = 1.80 ~ 2.00) was used to do further study. We chose MGI DNBSEQ-T7 pair-end sequencing (PE150 mode) to do resequencing project. For pair-end sequencing, at least 3 μg genomic DNA was used for sequencing library construction for each sample. Paired-end libraries with insert sizes of ~ 450 bp were prepared following Illumina’s standard genomic DNA library preparation procedure. Purified genomic DNA is sheared into smaller fragments with a desired size by Covaris, and T4 DNA polymerase was applied to generate blunt ends. After adding an ‘A’ base to the 3' end of the blunt phosphorylated DNA fragments, adapters are ligated to the ends of the DNA fragments. The desired fragments can be purified through gel-electrophoresis, then selectively enriched and amplified by PCR. The index tag could be introduced into the adapter at the PCR stage as appropriate followed by a library quality test. At last, the quantified pair-end library would be used for MGI DNBSEQ-T7 sequencing (150 bp*2, Shanghai BIOZERON Co., Ltd).

## Coverage analysis

The raw data were filtered using fastp [21], and the clean reads were aligned to the genome of our assembled genome with repetitive sequences masked using BWA v0.7.17-r1188 with default parameters [22]. The resulting output files were converted and sorted using SAMtools v0.1.19 4 [23]. We selected the genome reference of *T. vachellii* with accession number of GCA_033026395.1, the genomic coverage for each chromosome in each sample was calculated using BEDTools v2.30.0 with 10 kb sliding windows [24], and the read coverage has been standardized, with a value of 0.5 for one chromosome and a total value of 1 for a pair of homologous chromosomes. For ZZ/XX, the value is 1, while for a single Z/X or W/Y (female), the value is 0.5. For each chromosome, we calculated the fold change in coverage between males and females as log2 (average male coverage)—log2 (average female coverage).

## SNP density

To analyze SNP density, we used the mapping files obtained from the previous step. After marking duplicates, we called single-nucleotide polymorphic (SNP) sites using the GATK v4.2.0 joint calling pipeline [25]. To filter the variants, we applied the following parameters: "QD < 2.0 || FS > 60.0 || MQRankSum < -12.5 || RedPosRankSum < -8.0 || SOR > 3.0 || MQ < 40.0". The ratio of female to male SNP density was calculated for 10 kb sliding windows, and then the fold change in SNP density between males and females was calculated as log2 (sum male SNPs)—log2(sum female SNPs) for each chromosome. We plotted the read coverage and SNP density using custom Python scripts.

## Analysis of *FST*, PCA and LD

The biallelic SNPs were filtered with following parameters: –maf 0.05 –min-meanDP 4 –max-missing 0.9 using VCFtools 0.1.16 [26], *F*_ST_ was calculated in 10 kb sliding windows, treating the female offspring and male offspring as two separate populations.

We extracted the SNPs from the chromosome 3, and Principal component analysis (PCA) of was performed using PLINK v1.90b6.21 [27]. To assess LD decay of the chromosome 3 from males and females, the LD coefficient (*r*^2^) between pairwise SNPs within a 500 kb window was calculated using PopLDdecay v3.43 [28]. We used SNPeff v5.2c [29] to select the SNPs that are heterozygous in the males (XY) but homozygous in the females (XX), and LD heatmaps were generated using LDBlockShow v1.36 [30].

## Genome synteny analysis

Synteny analysis of the *T. vachellii* genome (GCA_033026395.1) with the genomes of.

*T. fulvidraco* (GCA_023638525.1) and *H. wyckioides* (GCF_019097595.1) was analyzed to identify chromosome structural changes among the three species*.* MCScanX was used to identify blocks of collinearity using paralog gene pairs [31].

## Estimation of synonymous (***d***_S_) and nonsynonymous mutation (***d***_N_)

To estimate the *d*_S_ between X chromosome and autosomes (except for chromosome 1 and 22) in *T. vachellii*, the coding sequences (CDS) from the X and autosomes of *T. vachellii* and *T. fulvidraco* were extracted, respectively. Orthofinder v.2.5.4 was used to cluster the CDS sequences of each homologous chromosome, respectively [32], and the single-copy ortholog sequences were chosen for alignment by PRANK [33]. Then *d*_S_ and *d*_N_ of each gene was calculated by yn00 from the PAML package v4.9 [34]. Bootstrapping with 1000 repetitions was used to generate 95% confidence intervals, and significant differences were determined using Graphpad Prism v8.2.1.

## Supplementary Information

Supplementary Material 1

## Data Availability

The whole genome re-sequencing data of *T. vachellii* are available in the NCBI SRA under PRJNA1198185.
