# Supplementary material for: Whole-genome resequencing reveals chromosomal fusion-driven early stages of XY chromosomes evolution in the darkbarbel catfish (Tachysurus vachellii)
Source: Front Zool. 2025 Nov 18;22:36. doi: 10.1186/s12983-025-00588-w (PMC12625426; doi:10.1186/s12983-025-00588-w)
Supplement: Supplementary file 1 — Supplementary Material 1 [file 12983_2025_588_MOESM1_ESM.docx]

**Supplementary Table and Figure**

**Table S1 Statistics of resequencing data from male and female individuals**

| Sample_ID | Raw_Reads (M) | Raw_Bases (G) | Clean_reads (M) | Clean_Bases (G) | Q20 (%) | Q30 (%) |
| --- | --- | --- | --- | --- | --- | --- |
| C1 | 96.31 | 14.45 | 94.21 | 14.08 | 98.25 | 95.63 |
| C2 | 95.57 | 14.34 | 93.48 | 13.97 | 98.09 | 95.20 |
| C3 | 99.87 | 14.98 | 97.54 | 14.57 | 98.07 | 95.16 |
| C4 | 100.29 | 15.04 | 98.02 | 14.65 | 98.14 | 95.34 |
| C5 | 102.11 | 15.32 | 99.77 | 14.91 | 98.16 | 95.38 |
| C6 | 100.04 | 15.01 | 97.86 | 14.63 | 98.17 | 95.37 |
| C7 | 101.10 | 15.17 | 98.92 | 14.78 | 98.17 | 95.33 |
| C8 | 98.32 | 14.75 | 96.06 | 14.35 | 97.83 | 94.59 |
| C9 | 96.57 | 14.49 | 94.73 | 14.16 | 98.11 | 95.25 |
| C10 | 104.50 | 15.68 | 102.51 | 15.32 | 98.08 | 95.19 |
| C11 | 113.64 | 17.05 | 111.32 | 16.63 | 97.88 | 94.71 |
| C12 | 102.19 | 15.33 | 100.25 | 14.99 | 98.06 | 95.17 |
| C13 | 101.17 | 15.18 | 98.71 | 14.74 | 97.66 | 94.13 |
| C14 | 107.88 | 16.18 | 105.79 | 15.82 | 98.14 | 95.39 |
| C15 | 91.08 | 13.66 | 89.28 | 13.34 | 98.00 | 94.99 |
| C17 | 98.94 | 14.84 | 96.64 | 14.44 | 97.96 | 94.84 |
| C18 | 91.78 | 13.77 | 89.85 | 13.43 | 98.30 | 95.59 |
| C19 | 126.76 | 19.01 | 124.66 | 18.64 | 98.31 | 95.66 |
| X1 | 105.92 | 15.89 | 103.86 | 15.52 | 98.01 | 95.04 |
| X2 | 112.99 | 16.95 | 110.72 | 16.55 | 97.93 | 94.87 |
| X3 | 116.08 | 17.41 | 113.81 | 17.01 | 97.98 | 94.88 |
| X4 | 109.57 | 16.44 | 107.34 | 16.04 | 98.03 | 95.10 |
| X5 | 124.10 | 18.62 | 121.49 | 18.15 | 97.87 | 94.70 |
| X6 | 115.60 | 17.34 | 113.35 | 16.94 | 98.08 | 95.18 |
| X7 | 107.81 | 16.17 | 105.88 | 15.83 | 98.23 | 95.49 |
| X8 | 105.02 | 15.75 | 103.03 | 15.40 | 97.94 | 94.78 |
| X9 | 100.96 | 15.14 | 98.98 | 14.79 | 97.72 | 94.27 |
| X10 | 124.92 | 18.74 | 122.64 | 18.33 | 98.12 | 95.21 |
| X11 | 106.85 | 16.03 | 104.85 | 15.67 | 98.04 | 95.00 |
| X12 | 107.75 | 16.16 | 105.70 | 15.80 | 98.03 | 95.01 |
| X13 | 109.69 | 16.45 | 107.47 | 16.06 | 98.05 | 95.14 |

Note: M means million, G represents Gigabase

**Figure S1 *F*_ST_ analysis of sex chromosomes**


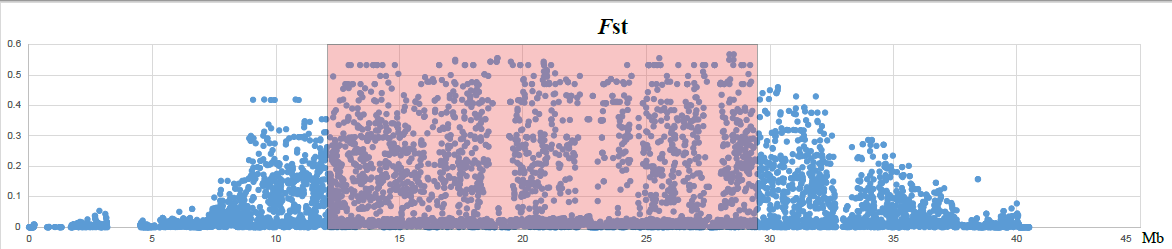


Note: The red shaded region indicates genomic segments with *F*_ST_ > 0.5, located at 12 Mb to 30 Mb.
